# Ion Conductivity in Salt-Doped Polymers: Combined Effects of Temperature and Salt Concentration

**DOI:** 10.1021/acsmacrolett.3c00757

**Published:** 2024-02-23

**Authors:** Alexandros J. Tsamopoulos, Zhen-Gang Wang

**Affiliations:** Division of Chemistry and Chemical Engineering, California Institute of Technology, Pasadena, California 91125, United States

## Abstract

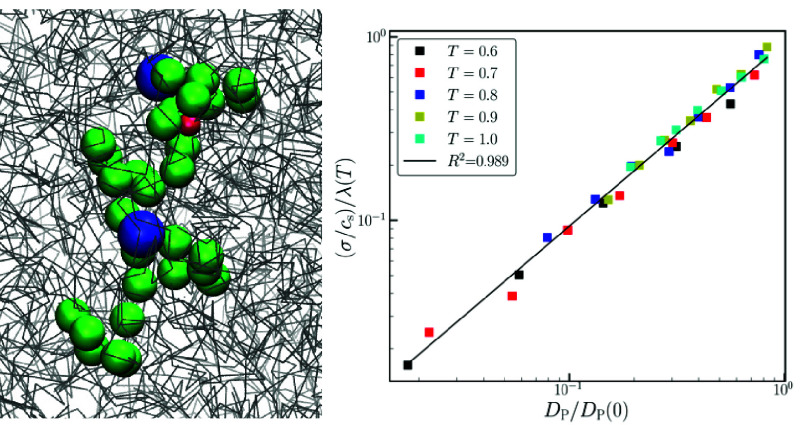

We construct a coarse-grained molecular dynamics model based on poly(ethylene oxide) and lithium bis(trifluoromethane)sulfonimide salt to examine the combined effects of temperature and salt concentration on the transport properties. Salt doping notably slows the dynamics of polymer chains and reduces ion diffusivity, resulting in a glass transition temperature increase proportional to the salt concentration. The polymer diffusion is shown to be well represented by a modified Vogel–Fulcher–Tamman (M-VFT) equation that accounts for both the temperature and salt concentration dependence. Furthermore, we find that, at any temperature, the concentration dependence of the conductivity is well described by the product of its infinite dilution value and a correction factor accounting for the reduced segmental mobility with increasing salt concentration. These results highlight the important role of polymer segmental mobility in the salt concentration dependence of ion conductivity for temperatures near and above the glass transition.

Electrolytes in energy storage devices, such as Li-ion batteries, typically consist of organic solvents and binary salts. Existing commercial energy storage devices use organic electrolytes, which come with safety and performance concerns,^1^ such as thermal runaway and electrolyte decomposition. Solvent-free polymer electrolytes (PEs), e.g., lithium salt-doped poly(ethylene)oxide (PEO), offer promising alternatives with low flammability, good mechanical stability, and suppression of the formation of Li dendrite growth.^2^ However, PEs suffer from low ion conductivity (σ ∼ 10^–4^ S cm^–1^) and low cation transference number (*t*_+_ ∼ 0.3).^3^ To overcome these performance bottlenecks, a comprehensive understanding of the ion transport mechanisms in these materials is necessary.

Seminal steps in this direction were taken by Borodin and Smith,^4^ who conducted molecular dynamics (MD) simulations with polarizable force fields. They proposed three ion transport mechanisms, namely, (1) polymer–ion co-diffusion, (2) ion motion along the polymer chain (intrachain), and (3) intersegmental ion hopping. Their study indicated that the dynamics of both cations and anions are coupled to polymer segmental relaxation. Further studies by Maitra and Heuer^5,6^ provided analytical expressions for the lithium ion diffusivity *D*_Li^+^_ in terms of the polymer chain length based on the three characteristic time scales of the proposed ion transport mechanisms. Additional MD studies by Molinari et al.^7^ suggested that polymer segmental relaxation is mainly responsible for the Li^+^ diffusion and that interchain hops are rare; however, this conclusion was based on simulations at much higher temperatures than the glass transition temperature of the system. While these studies focused on the mechanisms that determine Li^+^ diffusion, they did not explore the effect of salt concentration on the polymer mobility and on the glass transition temperature, factors that are crucial in determining ion transport.

Balsara and co-workers^8,9^ conducted quasi-elastic neutron scattering experiments to understand how salt concentration influences the ion conductivity of PEO doped with lithium bis(trifluoromethane) sulfonimide salt (PEO/LiTFSI). They found that the conductivity reaches a maximum with respect to salt concentration as a result of the competing effects of increased charge carriers and the slowdown of polymer segmental dynamics. This result was corroborated by the computer simulation study of Webb et al.^10^ for PEO doped with LiPF_6_, where addition of the lithium salt was shown to result in a global slowdown of chain dynamics.

An analogous trend to the concentration dependence of the ion conductivity was reported in terms of polymer host polarity. Ganesan and co-workers^11,12^ performed molecular dynamics simulations to examine how the polar nature of the polymer chain influences ion transport properties. It was found that, by increasing the dielectric permittivity of the polymer medium, the ion conductivity increases due to the weakened ion interactions. At higher dielectric constants, however, the enhanced polymer–polymer dipolar interactions impede the segmental relaxation dynamics, leading to a decrease of the ionic conductivity. They suggested that there is an intermediate dipole strength for which the ion conductivity is optimal.

In this Letter, we report results from coarse-grained molecular dynamics simulations aimed at exploring the combined effect of salt concentration and temperature on the ion transport in systems such as PEO doped with LiTFSI. We find that the polymer segmental dynamics are primarily responsible for the non-monotonic behavior of conductivity with salt concentration and propose a modified Vogel–Fulcher–Tamman (M-VFT) equation to describe the temperature and salt concentration dependence of the polymer segmental mobility that remains valid near the glass transition temperature.

We follow the simulation framework developed by Hall and co-workers^13−15^ to model lithium-salt-doped polymers (such as the PEO/LiTFSI system) in which the polymer dynamics is described by the Kremer–Grest model^16,17^ and the strong ion–polymer interaction is captured by a 1/*r*^4^ solvation potential. In order to capture glass transition and the effects of temperature on the segmental dynamics, we include an attractive interaction between non-bonded monomer beads.^18,19^ More details on the simulation methods and the mapping from coarse-grained to real units are provided in the Supporting Information.

As the chain dynamics is intimately related to the proximity to glass transition^18^ and lithium-salt doping is known to affect the chain dynamics,^8,10^ we first examine the dependence of glass transition temperature, *T*_g_, on salt concentration. A common method for determining *T*_g_ is by measuring the temperature dependence of the simulation volume under isobaric conditions.^20,21^ At the glass transition, there is a characteristic change in the slope of the volume when plotted as a function of the temperature (see Figure S1a). As shown in Figure 1, *T*_g_ increases with salt concentration and follows a straight line for the concentration range that we studied. Both the order of magnitude of the *T*_g_ shift and the nearly linear dependence on salt concentration are consistent with the experimental findings (see Figure S1b).^22^ Considering the simplicity of the coarse-grained model used in our work, the agreement is reasonable and reassuring. This behavior can be attributed to the increased ion–polymer complexation leading to restricted motion of the polymer backbone^10^ and to the decreased partial molar volume of the polymer (see Figure S1c). Further, it has been reported in previous studies^23^ that the ion–polymer interactions lead to dynamic cross-linking of the polymer chains, effectively increasing the apparent molecular weight and thus increasing *T*_g_.^24^

**Figure 1:**
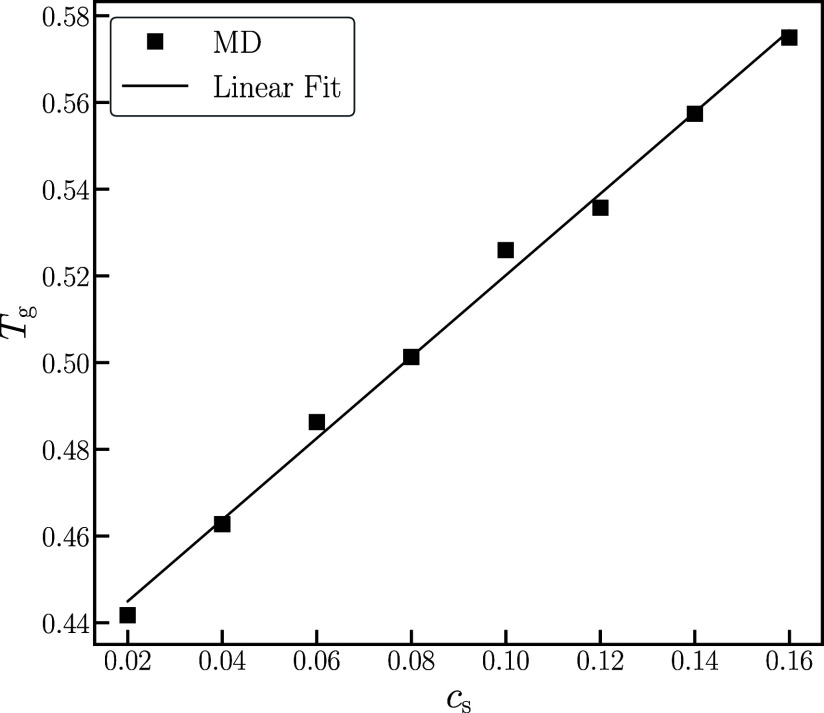
Glass transition temperature, *T*_g_, as a function of salt concentration, *c*_s_.

The most direct transport properties of the salt-doped polymer system are the self-diffusivities of the ions and center-of-mass self-diffusivity of the polymer, determined from
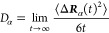
1where ⟨Δ***R***_α_(*t*)^2^⟩ is the mean-square displacement (MSD) of species α (α = +, −, and p) during time *t*. For the polymer, ***R***_p_(*t*) refers to the center-of-mass position of the chain. The salt concentration dependence of the diffusion of all species at *T* = 1.0 is shown in Figure S2. The diffusivities of all species decrease as *c*_s_ increases due to the strong ion–polymer coupling.^4^

Since the sum of the anion and cation diffusivities is proportional to the Nernst–Einstein conductivity, we examine its dependence on the salt concentration. Notably, while all of the diffusivities decrease with increasing salt concentration, Figure 2 shows that the ratio (*D*_+_ + *D*_–_)/*D*_p_ is relatively constant for each temperature. This result indicates that the ion diffusivities and polymer diffusivity have different temperature effects but similar salt concentration effects.

**Figure 2:**
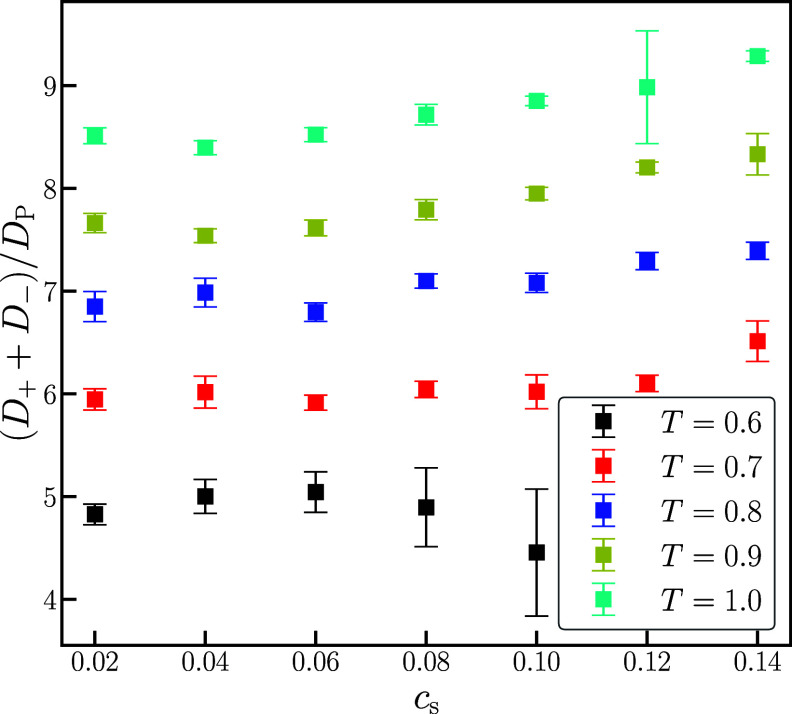
Ratio of the total ion diffusion over the polymer diffusion, (*D*_+_ + *D*_–_)/*D*_P_, as a function of salt concentration, *c*_s_.

For unentangled polymer melts, the polymer diffusivity is inversely proportional to the monomeric friction coefficient ζ. Previous studies have employed the Rouse model to examine the dynamics of polymer chains under lithium salt doping, nanoparticle doping, and concurrent salt and nanoparticle doping.^10,25,26^ As shown in Figure S4, the chain dynamics is well described by the Rouse modes for all the modes well above the glass transition. Near the glass transition, the high-*p* modes exhibit a notable deviation from the expected Rouse dynamics, but the low-*p* modes still follow the Rouse behavior. We thus take the lowest Rouse mode corresponding to the center-of-mass diffusion to define the friction coefficient via *D*_P_ = *kT*/*Nζ*. Mongcopa et al.^8^ have suggested that the friction coefficient follows an exponential dependence on salt concentration. Our results confirm this to be the case at high temperatures. However, at lower temperatures closer to *T*_g_, there is strong deviation from the exponential dependence; the friction instead becomes superexponential (see Figure S5).

Since the diffusion of a pure polymer melt close to *T*_g_ is well described by a Vogel–Fulcher–Tamman (VFT) temperature dependence, we propose a modified VFT (M-VFT) formula by taking into account the observed linear shift in the glass transition temperature with salt concentration and possible increase in the activation energy^27,28^
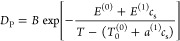
2where *B* is a prefactor, *E*^(0)^ is the activation energy in the pure polymer melt, and *T*_0_^(0)^ is the Vogel temperature of the pure polymer melt,^27^ typically taken to be 50 K below the glass transition temperature *T*_g_. *E*^(1)^ and *a*^(1)^ are coefficients for capturing the leading-order dependence on salt concentration in the activation energy and glass transition temperature, respectively.

In Figure 3, we show *D*_P_ in an Arrhenius plot for the 8 salt concentrations (including 0 salt) studied in our work. The symbols are the simulation data. *D*_P_ is seen to decrease with increasing salt concentration and decreasing temperature. The solid lines are the results of fitting. We find that, with a single set of fitting parameters, *B* = 0.01273, *E*^(0)^ = 1.295, *E*^(1)^ = 4.201, *T*_0_^(0)^ = 0.245, *a*^(1)^ = 1.249, eq 2 is able to describe all the simulation data well.

**Figure 3:**
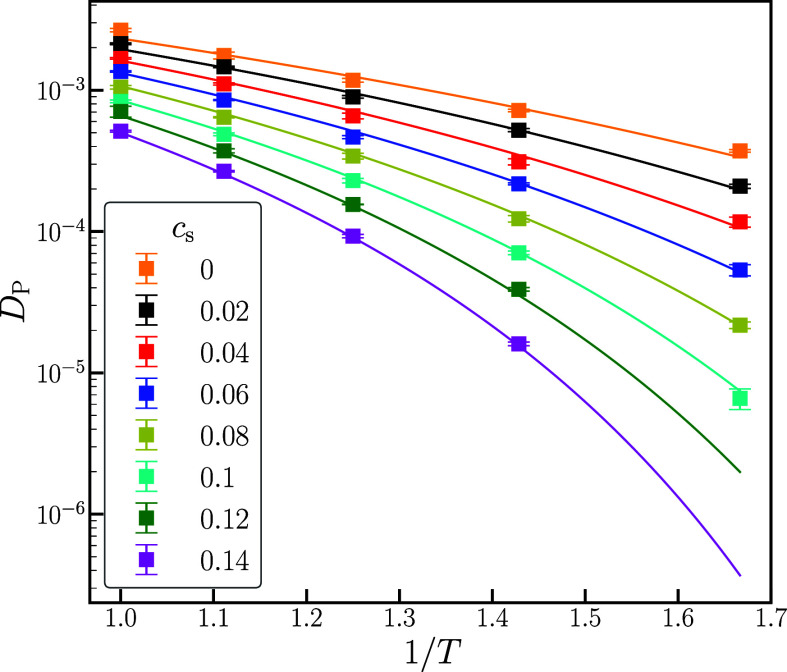
Polymer center of mass diffusivity, *D*_P_, as a function of 1/*T*, for various salt concentrations.

The ion conductivity is a complex function of ion–ion correlations and segmental dynamics. Typically in an electrolyte solution, increasing salt concentration increases both ion–ion correlations—in the form of ion pairing and ion clustering—and the friction coefficient or solution viscosity. Both factors contribute to a decrease in the ion mobility.^29,30^ However, for the PEO/LiTFSI system, the experimental work by Mongcopa et al.^8^ showed that the reduction of the conductivity is primarily due to the slowing down of the polymer segmental dynamics, implying that ion–ion correlation plays a relatively minor role in governing the conductivity.^31^ The physical reason for the weak ion–ion correlation in the PEO/LiTFSI system is likely due to the strong Li–PEO interaction which results in the seclusion of the Li^+^ ions from forming ion pairs and clusters with the bulky TFSI^–^ ions.^32^ In our coarse-grained model, the weakness of the ion–ion correlation can be gleaned from the radial distribution function of the polymer beads and anions from the cation (see Figure S3). To quantify the effects of the ion–ion correlation on the ion conductivity, we compare its value computed using the exact (Einstein) equation
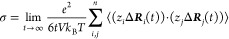
3with the approximate expression from using the ion diffusivities (the Nernst–Einstein equation)
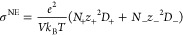
4In these expressions, *e* is the elementary charge, *V* is the simulation volume, *k*_B_ is the Boltzmann constant, *T* is the temperature, *z*_*i*_ is the ion valency, and Δ***R***_*i*_(*t*) is the displacement of particle *i* during time *t*. *N*_+_ and *N*_–_ are, respectively, the number of cations and anions, and *D*_+_ and *D*_–_ are the corresponding diffusivity. We note that, while the choice of reference frame can influence the cation transference number,^33,34^ the ion conductivity, which is the focus of this work, is independent of this choice.^35,36^

In Figure 4, we show the ion conductivity computed using eq 3 together with the Nernst–Einstein conductivity calculated using eq 4 as a function of salt concentration at the high temperature *T* = 1.0. With increasing salt concentration, the conductivity initially increases, reaches a maximum, and then decreases; this non-monotonic behavior is consistent with both earlier simulations and experiments.^4,8^ Within the error bars of the simulation data, the Nernst–Einstein conductivity is very close to the true conductivity, confirming that the ion–ion correlation is unimportant for this system. Both experimental and simulation studies have reported similar results,^4,11,37,38^ with inverse Haven ratios close to unity for this type of system.

**Figure 4:**
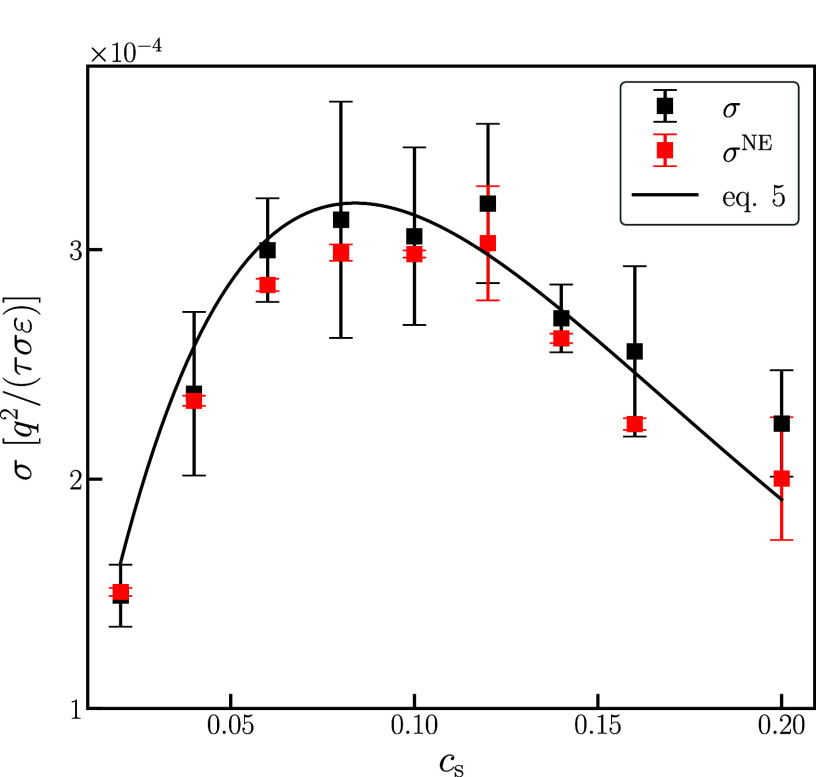
Ion conductivity, σ, as a function of salt concentration, *c*_s_, at temperature *T* = 1.0. We include the true conductivity, σ, the Nernst–Einstein conductivity, σ^NE^, and the conductivity based on eq 5.

Based on the fitted exponential dependence of the friction coefficient at high temperatures, ref (8) proposed the following expression for the salt concentration dependence of the conductivity
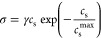
5where *c*_s_^max^ is obtained from fitting the friction coefficient. Our data at *T* = 1.0 shown in Figure 4 can be reasonably described by this expression. Later work by the same group^39^ found that, at lower temperatures, eq 5 could still fit the experimental data; however, the coefficients γ and *c*_s_^max^ were treated as purely fitting parameters *for each temperature* and no connection was made with the segmental friction.

Since the conductivity is well approximated by the Nernst–Einstein conductivity and since the ratio of the sum of the cation and anion diffusivities to the polymer diffusivity remains essentially independent of the salt concentration for a given temperature, we hypothesize that using the M-VFT equation (eq 2) for the polymer diffusivity would allow a unified description of the conductivity for any temperature and salt concentration. Thus, we propose
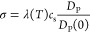
6where *D*_P_ is given by eq 2 and *D*_P_(0) = *D*_P_(*c*_s_ = 0). λ(*T*) corresponds to the specific conductivity at infinite dilution at temperature *T*, which can be directly calculated from performing simulations at very low salt concentrations or treated as a fitting parameter. In Figure S6, we show data for λ(*T*) obtained using both methods with good agreement. For our numerical fitting using eq 6, we adopt the value obtained from fitting.

In Figure 5a, we present the simulation data (symbols) for the ion conductivity as a function of salt concentration for various temperatures; in the same figure, we show the results of fitting using eq 6 (lines). As expected, the ion conductivity decreases as the temperature is decreased, while the characteristic non-monotonic dependence is found for all temperatures. The maximum in the conductivity shifts to lower concentrations as the temperature decreases, in agreement with experiments.^39^ All of these trends are captured by eq 6, which yields nearly quantitative agreement with the simulation data.

**Figure 5:**
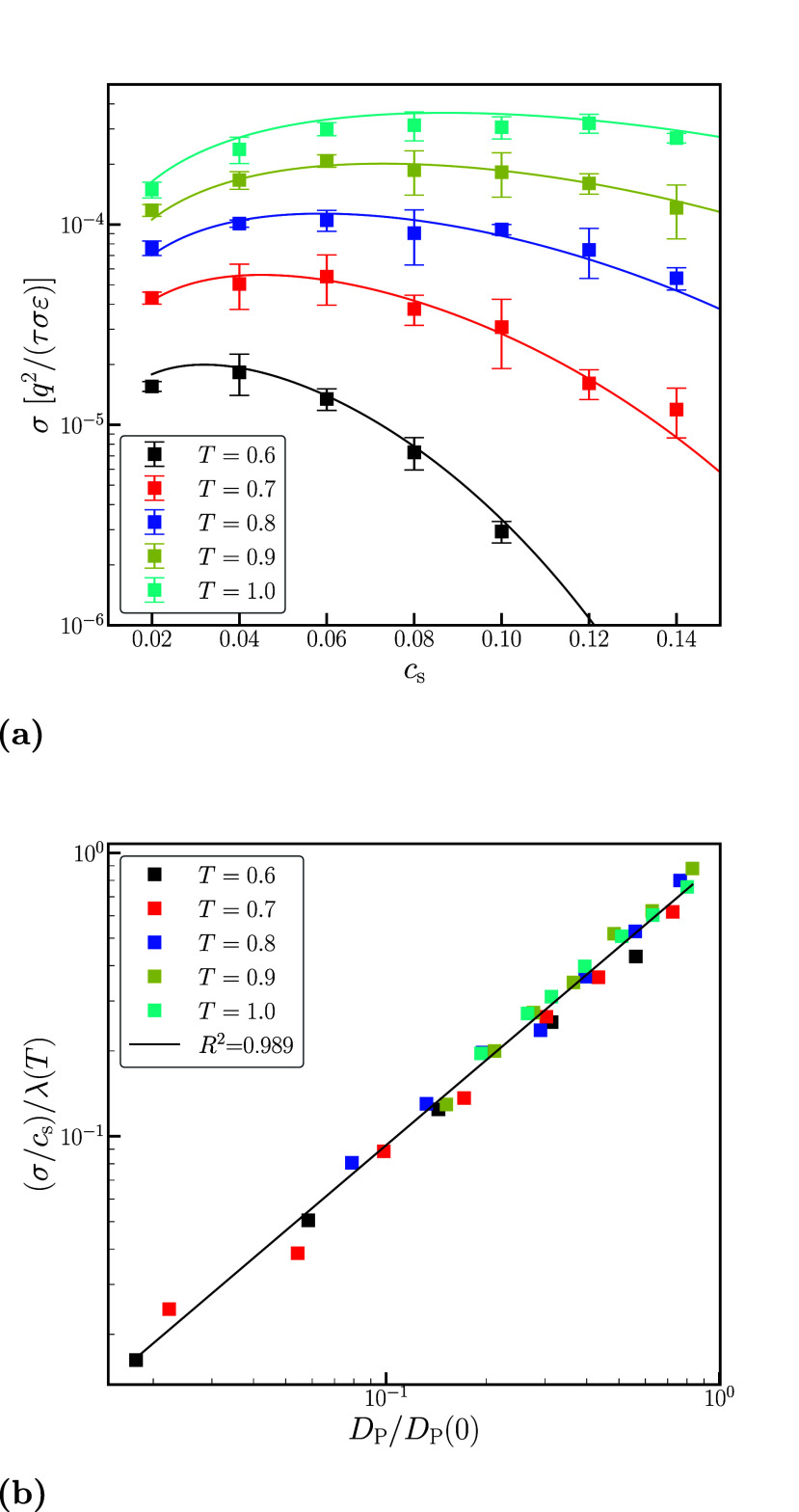
(a) Ion conductivity, σ, as a function of salt concentration, *c*_s_, for various temperatures, (b) Ratio of the specific conductivities correlates well with the ratio of polymer center-of-mass diffusivity.

As a global measure of the quality of fitting the data using eq 6, in Figure 5b, we plot the ratio of the specific conductivity σ/*c*_s_ to its infinite dilution value λ(*T*) vs the ratio of the polymer diffusivity *D*_P_/*D*_P_(0) on a log–log scale. All of the data fall on the same curve, with an *R*-squared value of 0.989. This result is a strong indication that the salt concentration dependence in the ion conductivity is governed by the segmental dynamics of the polymer for all the temperatures and salt concentrations examined in our work.

In the literature, the temperature dependence of the ion conductivity in amorphous polymeric materials is widely fitted to the VFT equation^40−42^
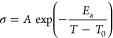
7where *A* and *E*_a_ are both fitting parameters and the Vogel temperature *T*_0_ is typically taken to be 50 K below *T*_g_, or treated as a fitting parameter. In Section 6 of the Supporting Information, we show the simulation data for the conductivity and the VFT fitting as a function of 1/*T* for different salt concentrations. While VFT provides a good fit to simulation data, we emphasize that the VFT parameters have to be fitted *for each salt concentration*, and their resulting dependence on the salt concentration exhibits behaviors that are difficult to justify on physical grounds, as shown and discussed in the Supporting Information. We believe the unusual behaviors of these fitting parameters with salt concentration, also reported in experimental studies,^28^ raise doubt about the physical basis in the VFT for describing the conductivity data in salt-doped polymers.

In summary, by examining the effects of salt concentration on the glass transition of the polymer, we propose a modified VFT equation for the polymer segmental mobility. Using the M-VFT equation, we provide a simple formula, eq 6, that accurately captures the conductivity data for all the temperatures and salt concentrations studied in our simulation and affords a straightforward physical interpretation. The complex concentration and temperature dependence in eq 6 cannot be captured by either eq 5 or eq 7 without using parameters that must be fitted for each temperature or concentration and that lack consistent physical interpretation. Interestingly, while lowering the temperature and increasing the salt concentration both slow down the segmental dynamics, we note that eq 6 does not follow a simple salt–temperature superposition as found in other systems such as ionomers.^43^ A possible reason for this difference may be that the salt concentration in the salt-doped polymer systems has a different role from that of the temperature, in that it alters the activation energy in the VFT equation, in addition to changing *T*_g_. Our study highlights the nontrivial synergistic effects of salt concentration and temperature on the segmental dynamics of polymers and ion conductivity, and the role of the glass transition temperature, in salt-doped polymer systems.

## Supporting Information Available

Simulation details, determination of the glass transition, ion–polymer coordination, Rouse-mode analysis and friction coefficient, specific conductivity at infinite dilution, and VFT analysis (PDF)
