# Supplementary material for: Ion Conductivity in Salt-Doped Polymers: Combined Effects of Temperature and Salt Concentration
Source: ACS Macro Lett. 2024 Feb 23;13(3):322–7. doi: 10.1021/acsmacrolett.3c00757 (PMC10956493; doi:10.1021/acsmacrolett.3c00757)
Supplement: Supplementary file 1 — mz3c00757_si_001.pdf [file mz3c00757_si_001.pdf]

Supporting Information: Supporting Information: Ion Conductivity in Salt-Doped Polymers: Combined Effects of Temperature and Salt Concentration

Alexandros J. Tsamopoulos and Zhen-Gang Wang*

Division of Chemistry and Chemical Engineering, California Institute of Technology, Pasadena, California 91125, United States

E-mail: zgw@caltech.edu

Contents

S1 Simulation Details **S-2**

S2 Determination of the Glass Transition **S-4**

S3 Ion–Polymer Coordination **S-6**

S4 Rouse-Mode Analysis and Friction Coefficient **S-7**

S5 Specific Conductivity at Infinite Dilution **S-9**

S6 VFT Analysis **S-10**

References **S-12**

S1 Simulation Details

Our simulations include 400 polymer chains, each with 30 neutral monomer beads and are conducted using Lennard–Jones (LJ) units. We choose the LJ parameter ϵ to correspond to $k_B T$ at $T = 400$K. Thus, the reduced temperature $T = 1.0$ corresponds to $T = 400$K. This correspondence yields a glass transition temperature that falls within the known values of the glass transition temperature for PEO[S1]. Following previous studies,[S2] the salt concentration mapping from LJ units to real units is $2c_{\mathrm{s}}^{\mathrm{LJ}} = c_{\mathrm{s}}^{\mathrm{real}} \equiv c_{\mathrm{s}}$, where c_{s} is defined as the ratio $[\mathrm{Li}^+]/[\mathrm{EO}]$. To compare with experiments, we report the salt concentration in real units. The length scale σ is taken as 0.7nm. All particles have the same mass $m = 1.0$.

All particles interact via the Lennard–Jones potential:

$$U_{\mathrm{LJ}}(r_{\mathrm{ij}}) = \begin{cases} 4\epsilon_{\mathrm{ij}} \left[\left(\frac{\sigma_{\mathrm{ij}}}{r_{\mathrm{ij}}}\right)^{12} - \left(\frac{\sigma_{\mathrm{ij}}}{r_{\mathrm{ij}}}\right)^{6} - \left(\frac{\sigma_{\mathrm{ij}}}{r_c}\right)^{12} + \left(\frac{\sigma_{\mathrm{ij}}}{r_c}\right)^{6} \right], & r_{\mathrm{ij}} \le r_{\mathrm{c}} \\ 0, & r_{\mathrm{ij}} > r_{\mathrm{c}} \end{cases} \tag{1}$$

where r_{ij} is the distance between two particles. We choose the same LJ interaction energy between all pairs $\epsilon_{\mathrm{ij}} = \epsilon$ and set σ_{ij} to be the mean particle size $\sigma_{\mathrm{ij}} = (\sigma_{\mathrm{i}} + \sigma_{\mathrm{j}})/2$. To approximate the size difference between the Li^+ cation, TFSI^- anion, and EO monomers in the PEO/LiTFSI system, we choose the polymer bead size to be 1.0σ, the cation diameter $\sigma_+ = 0.4\sigma$, and the anion diameter $\sigma_- = 1.6\sigma$. For the ion–ion, ion–monomer and bonded monomer–monomer interactions, the cutoff distance is $r_{\mathrm{c}} = 2^{1/6}\sigma_{\mathrm{ij}}$, corresponding to the repulsive Weeks–Chandler–Andsersen potential[S3]. For non-bonded monomer–monomer interactions we choose $r_{\mathrm{c}} = 2.0\sigma$, so that the interaction includes the attractive portion of the potential, which is essential to reproduce the glass transition phenomenon.[S4]

The chain connectivity is modeled using the finitely extensible nonlinear elastic potential (FENE)[S5,S6] between consecutive beads along the chain backbone:

$$U_{\mathrm{FENE}}(r_{\mathrm{ij}}) = -\frac{1}{2} K R_0 \ln\left(1 - \frac{r_{\mathrm{ij}}^2}{R_0^2}\right) \tag{2}$$

We choose the standard values $K = 30\epsilon/\sigma^2$ for the spring constant, and $R_0 = 1.5\sigma$ for the cutoff radius.

The strong ion–ether oxygen interaction in the lithium salt-doped PEO systems is captured by a solvation potential proposed by Hall and coworkers[S7,S8]:

$$U_{\mathrm{SOLV}}(r_{\mathrm{ij}}) = \begin{cases} -S_{\mathrm{ij}} \left[\left(\frac{\sigma_{\mathrm{ij}}}{r_{\mathrm{ij}}} \right)^4 - \left(\frac{\sigma_{\mathrm{ij}}}{r_{\mathrm{c}}} \right)^4 \right], & r_{\mathrm{ij}} \leq r_{\mathrm{c}} \\ 0, & r_{\mathrm{ij}} > r_{\mathrm{c}} \end{cases} \tag{3}$$

Following these authors, we choose $S_{\mathrm{ij}} = 4.33$ and $r_{\mathrm{c}} = 5.0\sigma$.

Ion–ion interactions are described by the Coulomb potential

$$U_{\mathrm{COUL}}(r_{\mathrm{ij}}) = \frac{q_{\mathrm{i}} q_{\mathrm{j}}}{4\pi\varepsilon_0 \varepsilon_{\mathrm{r}} r_{\mathrm{ij}}} \tag{4}$$

where q_{i} is the charge of ion i, ε_0 is the vacuum permittivity, and ε_{r} is the dielectric constant of the polymer host medium. For the temperature range studied we choose constant $\varepsilon_r = 7.5$, corresponding to the dielectric constant of PEO.[S2]

All simulations were performed on the LAMMPS platform.[S9] We used the velocity-Verlet algorithm with a time step of 0.005τ; where τ is the LJ time defined by $\sigma\sqrt{m/\epsilon}$. The Coulomb interactions were evaluated using a particle–particle particle–mesh Ewald solver.[S10] To control the temperature and pressure of the system we use the Nosé–Hoover barostat and thermostat,[S11] with a damping constant of 1.0τ. The pressure is set to $p = 0.0\varepsilon\sigma^{-3}$.[S12] For all our simulations, we prepared fully equilibrated systems at $T = 1.0$ for the range of salt concentrations studied. To examine the temperature dependence and glass transition, we took these equilibrated configurations for the different salt concentrations at $T = 1.0$ and applied a constant cooling rate[S13] $\Delta T/\Delta t = -0.5 \times 10^{-7}/\tau$ to the desired temperatures. Data were collected by further equilibration at each of the desired temperature values. The results were obtained by block averaging with at least four statistically uncorrelated blocks, where the polymer and ions reached the diffusive regime in each block.

S2 Determination of the Glass Transition

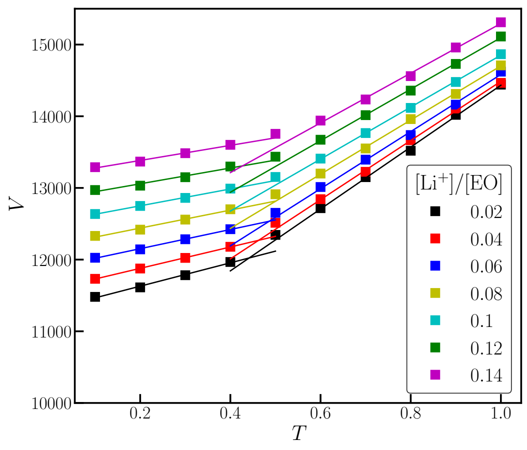

(a)

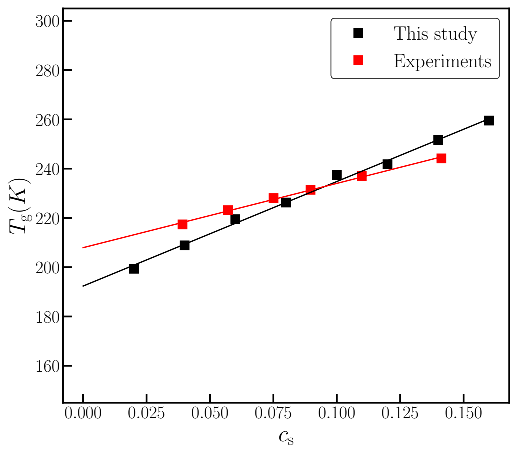

(b)

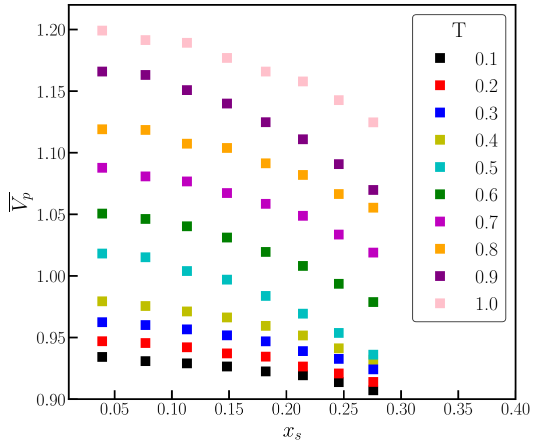

(c)

Figure S1: (a) System Volume, V, as a function of temperature, T, (b) Glass transition temperature T_{g}, as a function of salt concentration, c_{s}, from experiments[S1] and this work, (c) Polymer partial molar volume as a function of salt mole fraction

In Figure S1a, we show the system volume, V, as a function of temperature, T, for various salt concentrations. Each set of data exhibit two distinct slopes. By linearly fitting the volume data at low and high temperatures, we determine T_{g} from the crossing of the two straight lines.

To map the T_g of our simulation to that for the PEO/LiTFSI system, we invoke the requirement that our T_g in the absence of salt match the T_g of the neat polymer in experiments. Since T_g depends on the degree of polymerization, we use the Fox–Flory equation to determine the T_g corresponding to the chain length in our study, taking data from experiments at different chain lengths[S14]. We find that $T_g = 0.426$ in reduced units for our chain length without salt corresponds to $T_g = 192.3K$. Thus, in our study, a reduced temperature of $T = 1.0$ is mapped to $T \simeq 450K$. In Figure S1b, we show T_g in real units along with experimental data.[S1]. Recent simulation study has shown similar trend in the T_g dependence on salt concentration.[S15] Since our model parameters are not tuned to quantitatively reproduce properties of the PEO/LiTFSI system, and since the experiment used a different chain length, we do not expect quantitative agreement.

The increase in T_g with salt mole fraction can be attributed to the strong ion–polymer complexation, that leads to restricted motion of the polymer backbone, and to the decreased partial molar volume of the polymer as shown in Figure S1c. The monotonic behavior of $\overline{V}_p$ is found for all studied temperatures (below and above T_g).

S3 Ion–Polymer Coordination

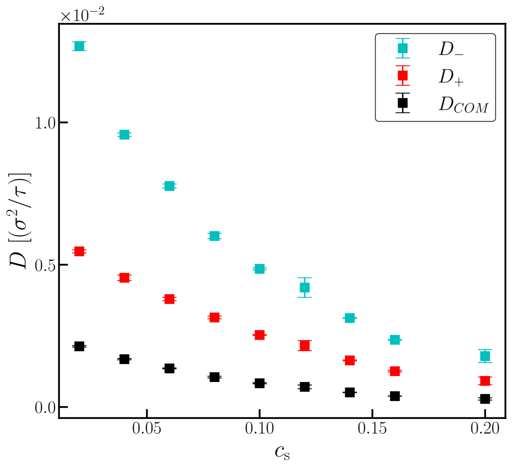

Figure S2: Self-diffusion coefficient, $\boldsymbol{D}$, as a function of salt concentration, c_s, for the anion, cation, and polymer center of mass, at temperature $\boldsymbol{T} = 1.0$.

In Figure S2, we show that the self-diffusion coefficients of the cation, anion, and polymer center–of–mass decrease with increasing salt concentration, c_s. This is attributed to the slowdown of the segmental dynamics due to the strong ion–polymer coupling.[S16]

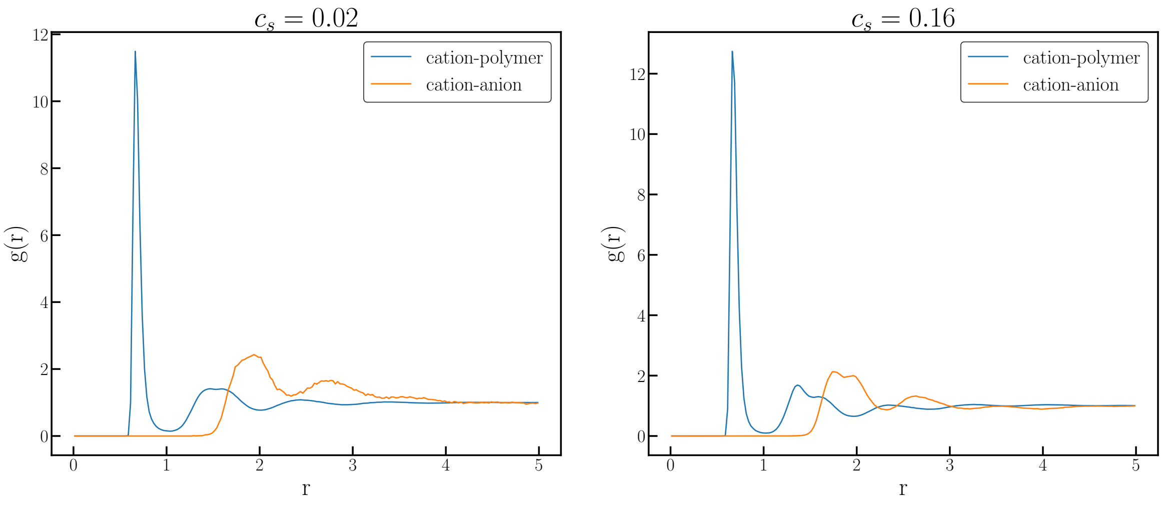

(a) (b)

Figure S3: The cation–polymer and cation–anion radial distribution functions at (a) $c_\mathrm{s} = 0.02$ and (b) $c_\mathrm{s} = 0.16$

In Figure S3, we show the cation–polymer and the cation–anion radial distribution func-

tion $g(r)$, for $c_s = 0.02$ and $c_s = 0.16$. For both concentrations, the peak height of the cation-polymer $g(r)$ is much higher than the peak height of the cation-anion $g(r)$. This result supports that the strong cation–polymer interaction leads to seclusion of the cation from forming ion pairs and clusters with the bulky anion in our model system.

S4 Rouse-Mode Analysis and Friction Coefficient

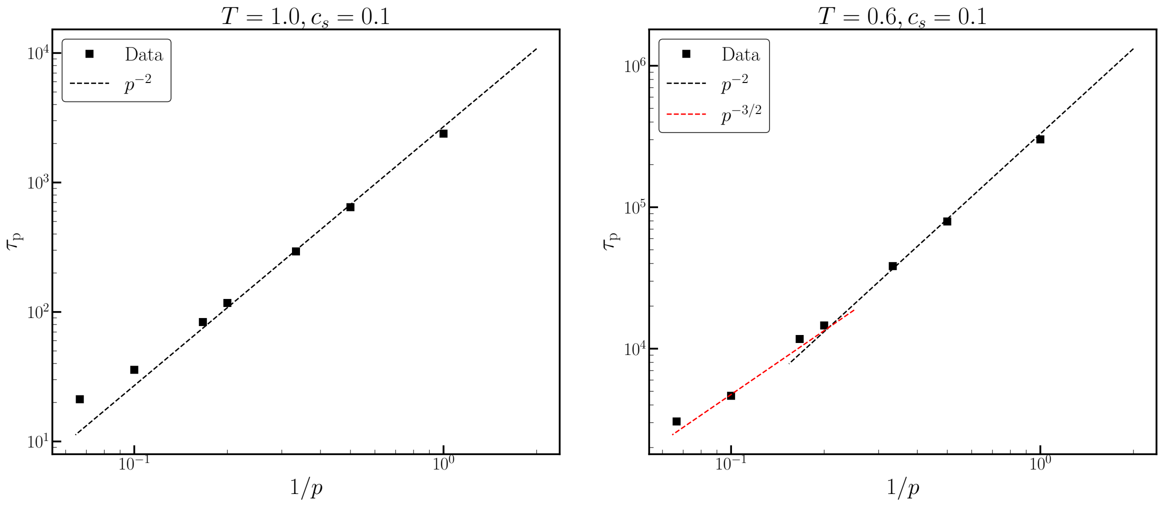

(a) (b)

Figure S4: Effective relaxation times plotted as a function of 1/p for (a) $T = 1.0$ and (b) $T = 0.6$

In Figure S4 we show the normal modes relaxation times, τ_p, at $T = 1.0$ and $T = 0.6$, for salt concentration $c_s = 0.1$. The normal mode analysis is described in Refs S17,S18. We find that at $T = 1.0$, τ_p follows the expected p^{-2} Rouse scaling. However, at $T = 0.6$ there is a clear deviation from the Rouse prediction. Specifically, there is a transition at some intermediate mode from p^{-2} to $p^{-3/2}$. The relationship between the location of the crossover and the temperature or salt concentration will be explored in future work.

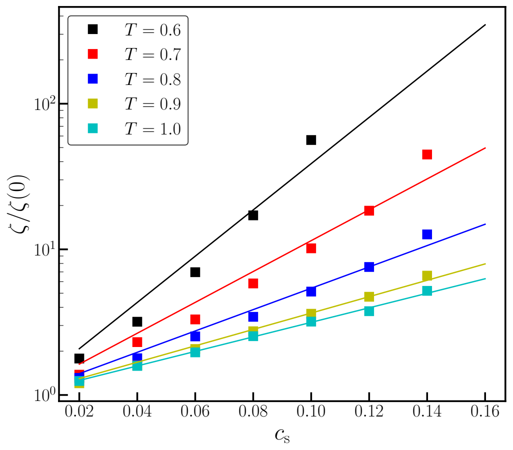

Figure S5: Normalized friction coefficient, $\zeta/\zeta(0)$, as a function of salt concentration, c_{s}, for various temperatures.

In Figure S5, we show the monomeric friction coefficient calculated from the Rouse model:

$$\zeta = \frac{k_{\mathrm{B}}T}{ND_{\mathrm{P}}} \tag{5}$$

All results are normalized by the friction coefficient of the pure polymer melt. With decreasing temperature, the salt concentration dependence of ζ changes from exponential to super-exponential. Due to this deviation, in the main text we have quantified the segmental dynamics using the modified-VFT equation for the polymer diffusion coefficient, instead of an exponential dependence on salt concentration for ζ.

S5 Specific Conductivity at Infinite Dilution

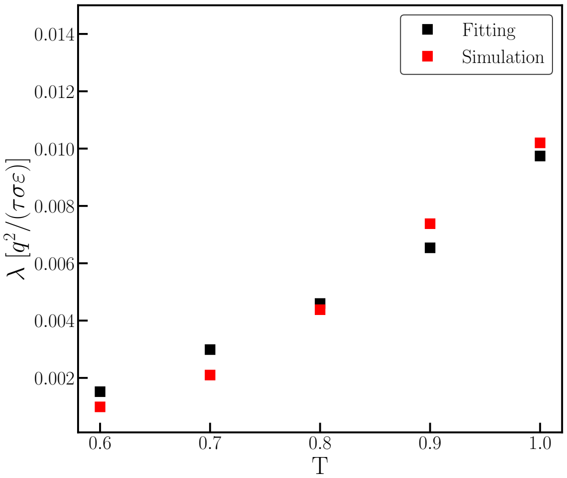

Figure S6: Specific Conductivity at infinite dilution as a function of temperature, calculated from fitting eq. 6 and conducting simulations at very low concentration

In Figure S6, we show the specific conductivity at infinite dilution, λ, as a function of temperature. We determine λ by fitting our conductivity data to eq. 6, and by conducting molecular simulations at very low salt concentrations ($c_s = 0.00016$). We find good agreement between the two methods for all the studied temperatures.

S6 VFT Analysis

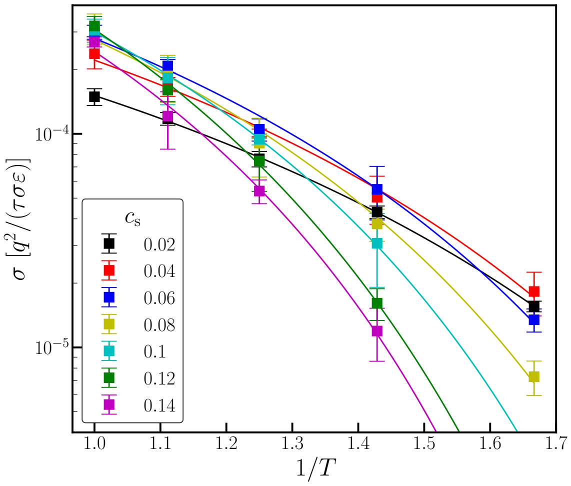

Figure S7: Ionic conductivity as a function of 1/T for various salt concentrations. The curves are results from fitting using scheme 1; the results are indistinguishable from those using schemes 2 and 3.

In Figure S7, we present the ionic conductivity as a function of $1/T$ for various salt concentrations. The symbols are the simulation data, and the lines are the results of fitting using the VFT equation:

$$\sigma = A\exp\left(-\frac{E_a}{T - T_0}\right) \tag{6}$$

where A is a prefactor commonly associated with the charge carrier concentration, E_a is a pseudoactivation energy related to the segmental relaxation, and T_0 is the equilibrium glass transition temperature, typically taken 50 K below T_g.[S19–S21] We fit our simulation data using 3 different schemes: (1) We consider $T_0 = T_g - 0.125$, where T_g is the glass transition temperature shown in Fig. 1 of the main text and 0.125 corresponds to 50 K in reduced units; (2) we consider T_0 to be predetermined from fitting the polymer diffusivity in eq. 6 of the main text; (3) T_0 is taken as a free fitting parameter. In all methods, A and E_a are considered fitting parameters.

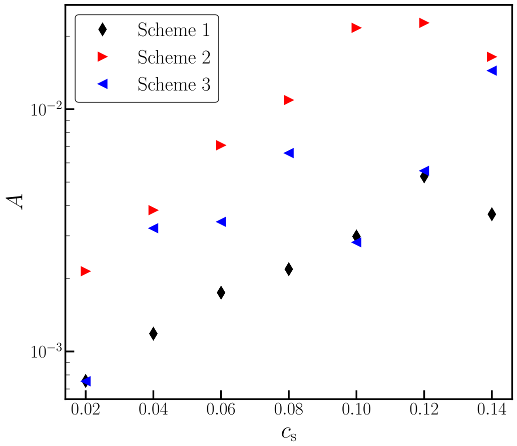

(a)

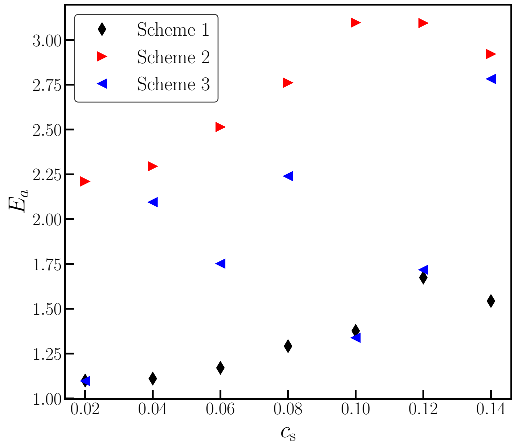

(b)

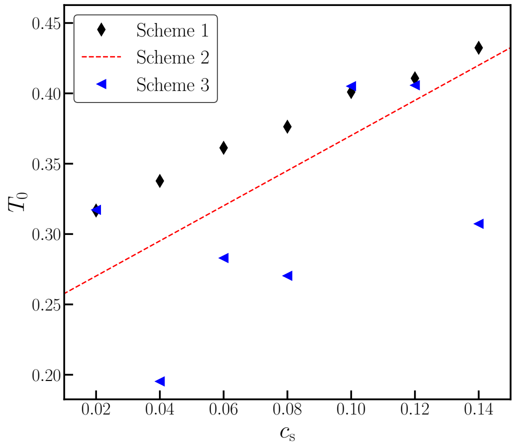

(c)

Figure S8: VFT parameters, (a) A (b) E_a and (c) T_0 calculated as a function of salt concentration, c_{s}, using the three different fitting methods.

In Figure S8, we show the parameters A, E_a, and T_0 as function of salt concentration, c_{s}. We observe that for fitting schemes 1 and 2, both A and E_a exhibit a non-linear and non-monotonic dependence on c_{s}. The non-monotonic behavior in both A and E_a are difficult to justify on physical grounds, given that our model does not have strong ion–ion correlations. Fitting scheme 3 yields a rather irregular dependence of T_0 on c_{s}, as shown in Figure S8c. The fitted values of T_0 fails to have any meaningful connection to the glass transition temperature, and likewise we find unphysical fluctuations in A and E_a. The fact that three different fitting

schemes with widely different values for the fitting parameters can yield nearly identical results to fit the conductivity data is strong evidence that the VFT fitting for the conductivity in salt-doped polymers lacks solid physical basis.

References

(S1) Lascaud, S.; Perrier, M.; Vallee, A.; Besner, S.; Prud'homme, J.; Armand, M. Phase Diagrams and Conductivity Behavior of Poly(ethylene oxide)-Molten Salt Rubbery Electrolytes. *Macromolecules* **1994**, *27*, 7469–7477, DOI: 10.1021/ma00103a034.

(S2) Shen, K.-H.; Hall, L. M. Ion Conductivity and Correlations in Model Salt-Doped Polymers: Effects of Interaction Strength and Concentration. *Macromolecules* **2020**, *53*, 3655–3668, DOI: 10.1021/acs.macromol.0c00216.

(S3) Weeks, J. D.; Chandler, D.; Andersen, H. C. Role of Repulsive Forces in Determining the Equilibrium Structure of Simple Liquids. *The Journal of Chemical Physics* **1971**, *54*, 5237–5247, DOI: 10.1063/1.1674820.

(S4) Morita, H.; Tanaka, K.; Kajiyama, T.; Nishi, T.; Doi, M. Study of the Glass Transition Temperature of Polymer Surface by Coarse-Grained Molecular Dynamics Simulation. *Macromolecules* **2006**, *39*, 6233–6237, DOI: 10.1021/ma052632h.

(S5) Grest, G. S.; Lacasse, M.; Kremer, K.; Gupta, A. M. Efficient continuum model for simulating polymer blends and copolymers. *The Journal of Chemical Physics* **1996**, *105*, 10583–10594, DOI: 10.1063/1.472978.

(S6) Kremer, K.; Grest, G. S. Dynamics of entangled linear polymer melts: A molecular-dynamics simulation. *The Journal of Chemical Physics* **1990**, *92*, 5057–5086, DOI: 10.1063/1.458541.

(S7) Brown, J. R.; Seo, Y.; Hall, L. M. Ion Correlation Effects in Salt-Doped Block Copolymers. *Physical Review Letters* **2018**, *120*, 127801, DOI: 10.1103/PhysRevLett.120.127801.

(S8) Shen, K.-H.; Fan, M.; Hall, L. M. Molecular Dynamics Simulations of Ion-Containing Polymers Using Generic Coarse-Grained Models. *Macromolecules* **2021**, *54*, 2031–2052, DOI: 10.1021/acs.macromol.0c02557.

(S9) Plimpton, S. Fast Parallel Algorithms for Short-Range Molecular Dynamics. *Journal of Computational Physics* **1995**, *117*, 1–19, DOI: 10.1006/jcph.1995.1039.

(S10) Brown, W. M.; Kohlmeyer, A.; Plimpton, S. J.; Tharrington, A. N. Implementing molecular dynamics on hybrid high performance computers – Particle–particle particle-mesh. *Computer Physics Communications* **2012**, *183*, 449–459, DOI: 10.1016/j.cpc.2011.10.012.

(S11) Glenn J. Martyna, D. J. T., Mark E. Tuckerman; Klein, M. L. Explicit reversible integrators for extended systems dynamics. *Molecular Physics* **1996**, *87*, 1117–1157, DOI: 10.1080/00268979600100761.

(S12) Hsu, H.-P.; Kremer, K. A coarse-grained polymer model for studying the glass transition. *The Journal of Chemical Physics* **2019**, *150*, 091101, DOI: 10.1063/1.5089417.

(S13) Li, W.; Olvera de la Cruz, M. Glass transition of ion-containing polymer melts in bulk and thin films. *Soft Matter* **2021**, *17*, 8420–8433, DOI: 10.1039/D1SM01098K.

(S14) Niedzwiedz, K.; Wischnewski, A.; Pyckhout-Hintzen, W.; Allgaier, J.; Richter, D.; Faraone, A. Chain Dynamics and Viscoelastic Properties of Poly(ethylene oxide). *Macromolecules* **2008**, *41*, 4866–4872, DOI: 10.1021/ma800446n, Publisher: American Chemical Society.

(S15) Fang, C.; Yu, X.; Chakraborty, S.; Balsara, N. P.; Wang, R. Molecular Origin of High Cation Transference in Mixtures of Poly(pentyl malonate) and Lithium Salt. *ACS Macro Letters* **2023**, *12*, 612–618, DOI: 10.1021/acsmacrolett.3c00041, Publisher: American Chemical Society.

(S16) Borodin, O.; Smith, G. D. Mechanism of Ion Transport in Amorphous Poly(ethylene oxide)/LiTFSI from Molecular Dynamics Simulations. *Macromolecules* **2006**, *39*, 1620–1629, DOI: 10.1021/ma052277v.

(S17) Hsu, H.-P.; Kremer, K. Detailed analysis of Rouse mode and dynamic scattering function of highly entangled polymer melts in equilibrium. *The European Physical Journal Special Topics* **2017**, *226*, 693–703, DOI: 10.1140/epjst/e2016-60322-5.

(S18) Kalathi, J. T.; Kumar, S. K.; Rubinstein, M.; Grest, G. S. Rouse mode analysis of chain relaxation in polymer nanocomposites. *Soft Matter* **2015**, *11*, 4123–4132, DOI: 10.1039/C5SM00754B, Publisher: The Royal Society of Chemistry.

(S19) Thomas, E. M.; Nguyen, P. H.; Jones, S. D.; Chabinyc, M. L.; Segalman, R. A. Electronic, Ionic, and Mixed Conduction in Polymeric Systems. *Annual Review of Materials Research* **2021**, *51*, 1–20, DOI: 10.1146/annurev-matsci-080619-110405.

(S20) Albinsson, I.; Mellander, B.-E.; Stevens, J. R. Ionic conductivity in poly(propylene glycol) complexed with lithium and sodium triflate. *The Journal of Chemical Physics* **1992**, *96*, 681–690, DOI: 10.1063/1.462453.

(S21) Ratner, M. A.; Nitzan, A. Conductivity in polymer ionics. Dynamic disorder and correlation. *Faraday Discussions of the Chemical Society* **1989**, *88*, 19–42, DOI: 10.1039/DC9898800019, Publisher: The Royal Society of Chemistry.
